# 

**Published:** 1854-04

**Authors:** 


*Short Comings.*—Most of the editorial notices intended for this number of the Journal have been crowded out by the press of other matter. The same circumstance has shortened very materially our Quarterly Summary. The missing paragraphs will, however, be found in our next number.

